# Nanoparticles for Brain Drug Delivery

**DOI:** 10.1155/2013/238428

**Published:** 2013-05-21

**Authors:** Massimo Masserini

**Affiliations:** Department of Health Sciences, University of Milano-Bicocca, Via Cadore 48, 20900 Monza, Italy

## Abstract

The central nervous system, one of the most delicate microenvironments of the body, is protected by the blood-brain barrier (BBB) regulating its homeostasis. BBB is a highly complex structure that tightly regulates the movement of ions of a limited number of small molecules and of an even more restricted number of macromolecules from the blood to the brain, protecting it from injuries and diseases. However, the BBB also significantly precludes the delivery of drugs to the brain, thus, preventing the therapy of a number of neurological disorders. As a consequence, several strategies are currently being sought after to enhance the delivery of drugs across the BBB. Within this review, the recently born strategy of brain drug delivery based on the use of nanoparticles, multifunctional drug delivery systems with size in the order of one-billionth of meters, is described. The review also includes a brief description of the structural and physiological features of the barrier and of the most utilized nanoparticles for medical use. Finally, the potential neurotoxicity of nanoparticles is discussed, and future technological approaches are described. The strong efforts to allow the translation from preclinical to concrete clinical applications are worth the economic investments.

## 1. Introduction

At the beginning of the third millennium, due to prolonged ageing, neurological disorders are growing, with a consequent high social impact due to their prevalence and/or high morbidity and mortality. For the purpose of calculation of estimates of the global burden of disease, the neurological disorders are included in two categories: neurological disorders within the neuropsychiatric category and neurological disorders from other categories. Neurological disorders within the neuropsychiatric category include epilepsy, Alzheimer and other dementias, Parkinson's disease, multiple sclerosis, and migraine. Neurological disorders from other categories include diseases and injuries which have neurological sequels such as cerebrovascular disease, neuroinfections, and neurological injuries.

Neurological disorders are an important cause of mortality and constitute 12% of total deaths globally. Among the neurological disorders, Alzheimer and other dementias are estimated to constitute 2.84% of the total deaths, while cerebrovascular disease constitute about 8% of the total deaths in high income countries in 2005 [1].

Presently, there are no effective therapies for many of them. Scientific and technological researches, from molecular to behavioral levels, have been carried out in many directions but they have not yet been developed in a truly interdisciplinary way, and a definitive response is still far to be prospected.

The immediate consequence of such condition is that several pathological disorders involving CNS remain untreatable. Examples of diseases include neurodegeneration (e.g., amyotrophic lateral sclerosis, Alzheimer's, Parkinson's, Huntington disease, and Prion Disease), genetic deficiencies (e.g., lysosomal storage diseases, leukodystrophy), and several types of brain cancer. Even if candidate drugs for therapy of such diseases may be already available in line of principle, they cannot be currently utilized because of their insignificant access to the central nervous system (CNS), due to the presence of the blood-brain barrier (BBB) [2] preventing the passage from blood to the brain.

## 2. The Blood Brain Barrier and Drugs

The BBB is a structure formed by a complex system of endothelial cells, astroglia, pericytes, and perivascular mast cells [3], preventing the passage of most circulating cells and molecules [4, 5]. The tightness of the BBB is attributed mainly to the vascular layer of brain capillary endothelial cells which are interconnected side-by-side by tight and adherens junctions. Tight junctions perform two functions: (i) they prevent the passage of small molecules and ions through the space between cells, so that their passage must occur by entering the cells (by diffusion or active transport). This pathway controls the type and amount of substances that are allowed to pass (ii) they prevent the movement of integral membrane proteins between the apical and basolateral membranes of the cell, so that each of the cell membrane surfaces preserves its peculiar functions, for example, receptor-mediated endocytosis at the apical surface and exocytosis at the basolateral surface. Three integral proteins are present at the tight junctions: occludin, claudins, and junctional adhesion molecules. The former two constitute the backbone of junction strands while junctional adhesion molecules are important for trafficking of T-lymphocytes, neutrophils, and dendritic cells from the vascular compartment to the brain during immune surveillance and inflammatory responses. Adherens junctions provide strong mechanical attachments between adjacent cells and are built from cadherins and catenins. The compact network of interconnections is conferring to the endothelial layer of the BBB a transelectrical resistance >1500 Ω cm^2^, which is the highest among all other endothelial districts. The compactness of the endothelial BBB layer precludes the passage across intercellular junctions (paracellular passage), limiting the possibility of exchanges between the two compartments virtually through passages transiting across the cellular body (transcellular passage).

However, the BBB is not only a mechanical fence but also a dynamic biological entity, in which active metabolism and carrier-mediated transports occur. Nutrients, including glucose, amino acids, and ketone bodies, enter the brain via specific transporters, whereas receptor-mediated endocytosis mediates the uptake of larger molecules, such as neurotrophins and cytokines [6–10]. The BBB prevents the brain uptake of most pharmaceuticals, with the exception of small hydrophilic compounds with a mass lower than 150 Da and highly hydrophobic compounds with a mass lower than 400–600 Da that can cross the membrane by passive diffusion [9]. The list of BBB-permeant drugs includes opiates (e.g., morphine, methadone, and meperidine), anxiolytics (diazepam, temazepam), SSRIs (paroxetine), and antipsychotics (chlorpromazine, promethazine) but does not include the majority of antibiotics and antitumorals.

As above said, the tightness of the BBB is preventing the pharmacological therapy of a number of neurological diseases. It should also be mentioned that a further obstacle for drugs crossing the cerebral capillary endothelium and entering the brain parenchyma is represented by the presence of the *P*-glycoprotein pump in the BBB, allowing the recognition of molecules necessary for the brain to enter the brain and the expulsion of other molecules, pharmaceuticals included.

Given such premises, it is conceivable that different approaches have been tried to allow pharmaceuticals to overcome the BBB. These explorative strategies have been ranging from invasive techniques, for example, through osmotic opening of the BBB [11], to chemical modifications of drugs in order to take advantage of physiological carrier-mediated transports, or exploiting the so-called “Trojan horse” technology, coupling BBB-impermeant pharmaceutical to molecules able to cross the barrier taking advantage of receptor-mediated transport systems [12].

Alternative routes of administration, able to reach the brain bypassing the BBB (e.g., intranasal), have been actively investigated, but in the line of principle they are facing the constraints of the limited surface of adsorption of the olfactory bulb, which is minimal compared to that of the BBB, thus, quantitatively reducing the possibility to reach the brain with relevant amounts of drugs [13].

## 3. Nanotechnology for Brain Drug Delivery

In the recent years, with the advent of nanomedicine, engineered tunable devices with the size in the order of billionth of meters have been proposed as an intriguing tool potentially able to solve the unmet problem of enhancing drug transport across the BBB [14, 15]. Among different devices, nanoparticles (NPs) technology is rapidly advancing. NPs are objects sized between 1 and 100 nm [16] that work as a whole unit in terms of transport and properties.

**Figure 1:**
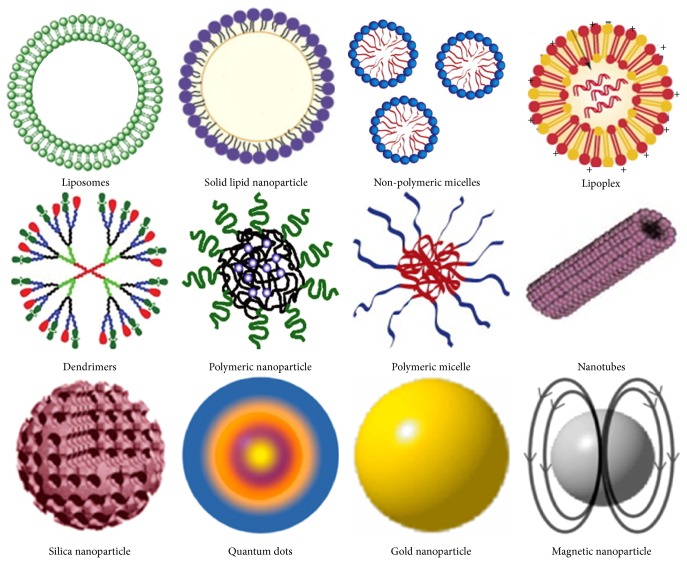
Different types of nanoparticles (NPs). Graphical representation of the most commonly used NPs for biomedical applications. NPs are typically by a size measuring not more than 100 nm and have significant potential for delivering drugs across the blood-brain barrier. The size of quantum dots is usually less than 10 nm.

The types of NPs that are more popular for biomedical applications are reported in Figure 1.

The reasons for this expectation are most of all linked to the possibility of NPs multifunctionalization, coupled to their ability to carry drug payloads, included BBB-impermeant drugs. In particular, the rationale of using NPs for brain drug delivery is that proper surface multifunctionalization may promote at the same time either their targeting of the BBB or the enhancement of its crossing. The possibility for BBB-impermeant drugs to reach the brain, when vehicled by NPs, is based upon the fact that their crossing of the barrier will depend completely on the physicochemical and biomimetic features of the NPs vehicle and will not depend anymore on the chemical structure of the drug, which is hindered inside the NPs.

What makes NPs even more attractive for medical applications is the possibility of conferring on them features such as high chemical and biological stability, feasibility of incorporating both hydrophilic and hydrophobic pharmaceuticals, and the ability to be administered by a variety of routes (including oral, inhalational, and parenteral) [17]. Moreover, NPs can be functionalized by covalent conjugation to various ligands (such as antibodies, proteins, or aptamers) to target specific tissues. The large surface-area-to-volume ratio of NPs permits multiple copies of a ligand to be attached and to dramatically increase their binding affinity via the multivalent functionalization [18].

**Figure 2:**
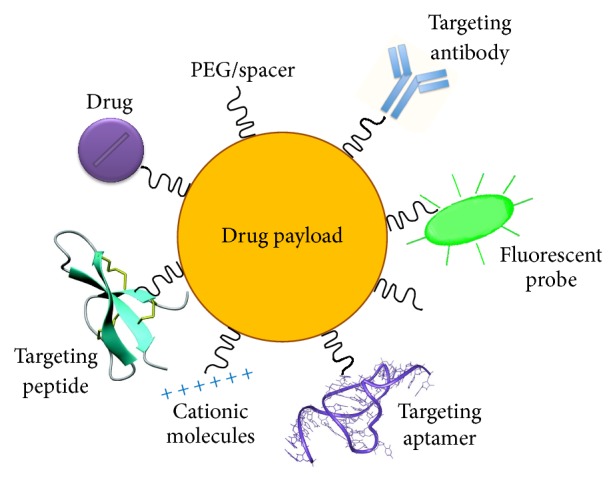
Multifunctionalized nanoparticles (NPs). Graphical representation of surface-modified NPs with drugs (incorporated within the core of NPs or conjugated to the surface), targeting molecules (antibodies, peptides, aptamers, and cationic molecules) for brain drug delivery, with PEG for stealthiness and with fluorescent probe as a tracer.

When designing NPs for clinical applications, it should be remembered that their systemic administration generates important modifications. In particular, the nonspecific interaction between the shell of NPs and many classes of proteins circulating in the bloodstream leads to the adsorption of opsonins on their surface, forming the so-called “corona.” These proteins substantially change the bare material properties determining the removal of NPs from circulation by the reticuloendothelial system, mainly located in spleen and liver. The most common approaches used for escaping RES are to formulate the particles with neutral surface charge, to coat their surface with different hydrophilic surfactants, such as polysorbates and polyethylene glycol (PEG), and to use small size nanoparticles (e.g., <80 nm) [19]. NPs with these features, called “stealth,” are able to avoid the reticuloendothelial system, to display long circulation time and stability in blood, and may be functionalized to successfully target and cross the BBB [20]. Finally, NPs should be nontoxic either for cells in the bloodstream or for healthy bystanding cells and should be biodegradable and biocompatible, noninflammatory and nonimmunogenic [21]. The possibilities of functionalization of MP for brain drug delivery are depicted in Figure 2.

It is also important to point out that the tailoring of NPs to enhance drug delivery to the brain does not necessarily imply their ability to cross the BBB themselves. It is predictable that NPs could play this role at least in two ways:

- by increasing the drug concentration inside, or at the luminal surface of BBB cells, establishing a local high concentration gradient between blood an brain, higher than that obtainable after systemic administration of the free drug. The gradient should then favor the enhanced passive diffusion of the drug. As for example, this task could be realized by synthesizing NPs functionalized to target brain capillary endothelial cells. This feature can be followed or not by their subsequent uptake from targeted cells [22];
- by moving themselves into the CNS, together with their drug cargo. As for example, this task can be realized enabling NPs targeting of brain capillary endothelial cells and their subsequent transcellular passage across the BBB [23].

For the completeness of this review, in the next section, the main features of NPs commonly utilized for medical purposes and already utilized, or promising candidates for brain drug delivery, will be described.

## 4. Nanoparticles for Medical Applications

### 4.1. Lipid-Based Nanoparticles

#### 4.1.1. Liposomes

Liposomes are the first generation of nanoparticulate drug delivery systems [24] and are constituted by one or more vesicular bilayers (lamellae) composed of amphiphilic lipids, delimiting an internal aqueous compartment. Usually, the liposomal lipid bilayer is composed of biocompatible and biodegradable lipids, present in biological membranes. Common liposome constituents are sphingomyelin, phosphatidylcholine, and glycerophospholipids. Cholesterol, an important component of cell membranes, is frequently included in liposome formulations because it decreases the bilayer permeability and increases the stability of the liposome *in vivo*. Liposomes are classified on the base of their size and the number of lamellae as follows (i) small unilamellar vesicles (SUV) with a size up to 100 nm and one bilayer, (ii) large unilamellar vesicles (LUV) with a size >100 nm and one bilayer, and (iii) multilamellar vesicles (MLV) that can reach a size of several *μ*m and made of many concentric lipid bilayers.

Liposomes have been largely utilized for brain drug delivery (for a review see [25]), for the treatment of cerebral ischemia [26], for delivery of opioid peptides [27], and brain tumours [28].

*Cationic Liposomes*. Cationic liposomes containing positively charged lipids have been developed and initially used as transfection vehicles, to deliver genetic material (e.g., DNA) into the cell, avoiding the lysosomal digestion. The most commonly utilized cationic lipid is 1,2-dioleoyl-3-trimethylammonium-propane (DOTAP), mixed with dioleoyl-phosphatidylethanolamine (DOPE). The cholesterol also increases the levels of transfection and can potentially reduce the destabilization of the liposomes in the presence of serum [29].

The interactions between cationic lipids and nucleic acids lead to the formation of structures, which are called “lipoplexes” [30]. Lipoplexes are typically formed by direct mixing between cationic liposomes and DNA solutions. Positively charged liposomes bind to negatively charged phosphate molecules on the DNA backbone through electrostatic interactions that are embedded and are shielded from the environment. Generally, complexes are formed with a slight excess positive charge to permit them to interact with the negatively charged cell surface. The cationic liposomes used are typically small before adding to DNA; however, complexes formed with DNA exhibit diameters that range from as small as 200 nm to structures as large as 2 *μ*m. Unlike liposomes, cationic liposomes undergo adsorptive-mediated endocytosis and internalization in endosomes. Upon acidification at pH 5 to 6, DOPE fuses and destabilizes the endosomal membrane, releasing its contents to the cytosol. Therefore, drugs could be vehicled into endothelial cells, similarly to DNA, enhancing their crossing of the barrier and reaching neurons. As a proof of this possibility, transfection of neuronal SH-SY5Y cells was achieved with the lipoplexes at a degree much higher than the degree obtained with the widely and commonly utilized transfectant Lipofectamine; cationic liposomes carrying a photoreactive drug resulted in a laser-stimulated cytotoxic effect on glioblastoma cells and showed the ability to improve brain drug delivery of paclitaxel (a mitotic inhibitor used in cancer chemotherapy) in rodents *in vivo* [31–33].

#### 4.1.2. Solid Lipid Nanoparticles

Solid lipid nanoparticles (SLN) are a stable lipid-based nanocarrier with a solid hydrophobic lipid core, in which the drug can be dissolved or dispersed [34]. They are made with biocompatible lipids such as triglycerides, fatty acids, or waxes. They are generally of small size (around 40–200 nm) allowing them to cross tight endothelial cells of the BBB and escape from the reticuloendothelial system (RES) [35].

During their fabrication the melted lipid, mixed with the drug, is commonly dispersed in an aqueous surfactant by high-pressure homogenization or microemulsification. The advantages of SLN are their biocompatibility, drug entrapment efficiency comparatively higher than other NPs, and the ability to provide a continuous release of the drug for several weeks [36]. Moreover, the composition of SLN can be controlled modifying their surface properties to target molecules to the brain and to limit RES uptake [37].

Several reports are available describing an enhanced drug delivery to the brain mediated by SLN. For instance, SLN carrying a calcium channel blocker drug, administered *i.v.* into rodent, showed that the drug was taken up to a greater extent by the brain and maintained high drug levels for a longer time compared to free drug suspension.

Wang et al. have reported the synthesis of 3′,5-dioctanoyl-5-fluoro-2-deoxyuridine (DO-FUdR) to overcome the limited access of the drug 5-fluoro-2,-deoxyuridine (FUdR) and its incorporation into SLN. The results indicated that DO-FUdR-SLN had brain targeting efficiency *in vivo* of about 2-fold compared to free FUdR. These authors report that SLN can improve the ability of the drug to penetrate through the BBB and is a promising drug targeting system for the treatment of central nervous system disorders [38, 39].

### 4.2. Polymer-Based Nanoparticles

#### 4.2.1. Polymeric Nanoparticles

Polymeric NPs are composed of a core polymer matrix in which drugs can be embedded [40–42], with sizes usually between 60 and 200 nm. A range of materials have been employed for delivery of drugs. In particular, in recent years some polymers have been designed primarily for medical applications and have entered the arena of controlled release of bioactive agents. Many of these materials are designed to degrade within the body. Most popular ones are polylactides (PLA), polyglycolides (PGA). poly(lactide-co-glycolides) (PLGA), polyanhydrides, polycyanoacrylates, and polycaprolactone. In spite of development of various synthetic and semi-synthetic polymers, also natural polymers such as chitosan can be utilized.

Also in the case of polymeric NPs, reports are available describing an enhanced drug delivery to the brain mediated by these devices. NPs made of PLGA embedding antituberculosis drugs (rifampicin, isoniazid, pyrazinamide, and ethambutol) for cerebral drug delivery were administered to mice, maintaining high drug levels for 5–8 days in plasma and for 9 days in the brain, much higher as compared with free drugs [43]. In Mycobacterium tuberculosis-infected mice, 5 doses of the NPs formulation (against 46 doses of conventional free drugs) resulted in undetectable bacteria in the meninges [44]. In another research [45], polybutyl-cyanoacrylate (PBCA) NPs were successfully utilized for delivery of functional proteins into neurons and neuronal cell lines.

#### 4.2.2. Polymeric Micelles

Polymeric micelles are formed by amphiphilic copolymers whose aggregation in aqueous media leads to spheroidal structures with a hydrophilic shell and a hydrophobic core and with a good grade of stability [36]. Stability can be improved by crosslinking between the shell or the core chains. Additional tunable features of polymeric micelles are the possibility to render them responsive to external stimuli (pH, light, temperature, ultrasound, etc.) [46], triggering a controllable release of entrapped drugs. One of the most utilized polymer is Pluronic type, block copolymer based on ethylene oxide and propylene oxide.

The possibility to use these NPs for brain drug delivery has been described. For instance, chitosan-conjugated Pluronic nanocarriers with a specific target peptide for the brain (rabies virus glycoprotein; RVG29) following *i.v.* injection in mice displayed *in vivo* brain accumulation either of a quantum dot fluorophore conjugated to the nanocarrier, or of a protein loaded into the carrier [47]. Other studies have shown an increased central analgesic effect of micellar-vehicled drug [48].

#### 4.2.3. Dendrimers

Dendrimers are branched polymers, reminding the structure of a tree. A dendrimer is typically symmetric around the core, and when sufficiently extended it often adopts a spheroidal three-dimensional morphology in water. A central core can be recognized in their structure with at least two identical chemical functionalities; starting from these groups, repeated units of other molecules can originate, having at least one junction of branching. The repetitions of chains and branching result in a series of radially concentric layers with increased crowding. The structure is therefore tightly packed in the periphery and loosely packed in the core, leaving spaces which play a key role in the drug-entrapping ability of dendrimers [49]. Poly(amidoamine), or PAMAM, is perhaps the most well-known molecule for synthesis of dendrimers. The core of PAMAM is a diamine (commonly ethylenediamine), which is reacted with methyl acrylate and then with another ethylenediamine to make the generation-0 PAMAM. Successive reactions create higher generations. Albertazzi et al. [50] showed that functionalization of PAMAMs dendrimers has a dramatic effect on their ability to diffuse in the CNS tissue *in vivo* and penetrate living neurons as shown after intraparenchymal or intraventricular injections.

Kannan et al. [51] showed that systemically administered polyamidoamine dendrimers localize in activated microglia and astrocytes in the brain of newborn rabbits with cerebral palsy, providing opportunities for clinical translation in the treatment of neuroinflammatory disorders in humans.

## 5. How NPs Can Cross the BBB

Many medicines are not able to reach the brain due to the lack of drug-specific transport systems through the BBB. The development of new strategies based on NPs to enhance the brain drug delivery is of great importance in the therapy and diagnosis of CNS diseases and it is based on the interactions between NPs and the BBB and on their intracellular traffic pathways.

### 5.1. Crossing the BBB without Functionalization

Although almost all nanomaterials fall into the class of BBB impermeable, some exceptions have been reported in recent years. For instance, gold and silica NPs have been shown to reach the brain and accumulate in neurons even in the absence of any specific functionalization, with a mechanism that substantially is still unknown. In the case of silica the results indicated that NPs administered to rodents via intranasal instillation entered into the brain and especially deposited in the striatum [52]. In the case of gold NPs precise particle distribution in the brain was studied *ex vivo* by X-ray microtomography, confocal laser and fluorescence microscopy [53]. The authors found that the particles mainly accumulate in the hippocampus, thalamus, hypothalamus, and the cerebral cortex. The same holds true for Titanium dioxide NPs that were found to cross the mice BBB particularly when smaller than 40 nm [54].

### 5.2. Adsorptive-Mediated Transcytosis

The concept of adsorptive-mediated transcytosis through the BBB was originally suggested by the observation that cationic proteins can bind the endothelial cell surface but also cross the BBB [55]. The mechanism, applied to NPs, is based on the proper functionalization of their surface allowing electrostatic interaction with the luminal surface of BBB. Given the presence of negative charges on endothelial cells [56] this interaction can be promoted by conferring a positive charge to the NPs surface.

Different procedures can be followed to realize this issue. A first possibility is to build up NPs made of components that are bearing a positive charged at physiological pH (7.4). This is the case of nanosized vesicles made of bolaamphiphilic molecules (amphiphilic molecules that have hydrophilic groups at both ends of an hydrophobic chain) that, following intravenous administration to mice, showed a marked accumulation of their encapsulated fluorescent cargo, whereas nonencapsulated probe was detected only in peripheral tissues but not in the brain [57]. In another investigation Jin et al. [58] used SLN made with lipids extracted from deproteinated lipoproteins and enriched with cationic cholesteryl hydrochloride and phosphatidyl-ethanolamine. The authors, after intravenous administration of such cationic NPs for the delivery of siRNA inhibiting c-Met expression, suppressed tumor growth without evident signs of systemic toxicity in an orthotopic xenograft tumor mice model of glioblastoma.

A second possibility is to functionalize the NPs surface with positively charged biomolecules combining their physicochemical features with biological activity. This is the case of cell-penetrating peptides (e.g., TAT peptides derived from HIV, gH625 derived from Herpes simplex virus type 1) and cationic proteins (e.g., albumin) [59] that have been extensively used for NPs decoration, facilitating the BBB passage of drugs [60]. As for example, Rao et al. [61] after administration of ritonavir via TAT-conjugated NPs demonstrated approximately an 800-fold higher level of drug in the brain when compared to that with free drug, with a remarkable enhancement of drug delivery. The authors suggested that TAT-conjugated polymeric NPs are first mobilized in the brain vasculatures, then transported to the brain parenchyma where they continue to release the drug.

Polymeric micelles having TAT molecules on the surface were successfully fabricated also to incorporate antibiotics and were able to cross the BBB [62]. In another study, Xu et al. [63] showed efficacy of TAT-polymeric NPs for the treatment of *C. albicans* meningitis in rabbits. Similar results are reported using another family of cell-penetrating peptides (SynB peptides RGGRLSYSRRRFSTSTGR) that were able to increase drug permeability across a cocultured BBB model, doubling drug transport *in vivo* in healthy mice [64].

Since the effect of cell-penetrating peptides could be invalidated by the rapid systemic clearance of functionalized NPs due to their positive charge, in a study dedicated to investigate this issue, Xia et al. [65] utilized penetratin, a peptide derived from Antennapedia protein (Drosophila), with relatively lower content of basic amino acids, to functionalize poly(ethylene glycol)-poly(lactic acid) (PLGA) NPs, fluorescently labeled with coumarin-6, and detected an increase of fluorescence in the rodent brain with respect to unfunctionalized NPs.

Xia et al. [65] also combined the use of cell-penetrating peptide to coumarin-6-loaded NPs for intranasal administration. The amount of fluorescent probe, detected in the rat cerebrum and cerebellum, was found to be more than 2-fold compared to that of coumarin carried by unfunctionalized NPs. Brain distribution analysis suggested that NPs after intranasal administration could be delivered to the central nervous system along both the olfactory and trigeminal nerves pathways.

The efficacy of cationic albumin to functionalize and promote NPs-carried drug delivery to the brain has been repeatedly reported [66, 67]. The property of cationic proteins to efficiently penetrate cells raises the question of the potential toxicity and immunogenicity of these proteins. The possible toxic effects include a generalized increase in vascular permeability since breakdown of BBB permeability and other vascular beds has been observed following intravenous injection of large amounts of cationic proteins but has not been seen when moderate amounts of these substances were administered [68].

### 5.3. Receptor-Mediated Transcytosis

One of the most recently applied strategies for drug delivery across the BBB endothelium using functionalized NPs is the one exploiting the transcytosis physiological mechanism of transport of macromolecules, relying on the presence of specific receptors on the luminal surface of cells. Transcytosis is the process by which extracellular cargo internalized at one plasma membrane domain (e.g., apical) of a polarized cell is transported via vesicular intermediates to the controlateral plasma membrane (e.g., basolateral). As for example, insulin, transferrin, apolipoproteins, and 2-macroglobulin are some of the proteins that reach the brain following the path across endothelial cells.

The rationale for exploiting this strategy with NPs is that the existing cellular mechanisms, handling macromolecular cargoes, may fit also to transport NPs after their functionalization to interact with the same receptors.

Transcytosis in endothelial cells starts with internalization of extracellular cargo into the cell by vesicular carriers. The cargo is subsequently processed via different pathways to appropriate intracellular organelles and recycled, degraded, or transcytosed to the contralateral side. Strong morphological and biochemical evidence suggest that the two membranes, apical and basolateral, are interconnected via vesicular intermediates involving multivesicular bodies or a system of endosomal vacuoles and tubule vesicles. Phosphoinositide 3-kinase, an enzyme increasingly demonstrated to play an important regulatory role in many vesicular trafficking events, also appears to regulate transcytotic traffic between the two endosomal compartments.

Transcytosis in endothelial cells starts with uptake either through clathrin-coated pits, by caveolae, or caveolae-like membrane domains [69] that will be described here below.

The first step of internalization through clathrin-mediated endocytosis is the binding of a ligand to a specific cell surface receptor. This results in the clustering of the ligand-receptor complexes in coated pits on the plasma membrane, which are formed by the assembly of cytosolic coat proteins; the main assembly units being clathrin, which form a polygonal lattice in the surface of the membrane; and adaptor protein complexes, which mediate the assembly of the clathrin lattice on the membrane. The coated pits then invaginate and pinch off from the plasma membrane to form intracellular clathrin-coated vesicles. The clathrin coat then depolymerizes, resulting in early endosomes, which fuse with each other or with other preexisting endosomes to form late endosomes that further fuse with lysosomes. Vesicular trafficking after CME is controlled by the action of small GTPases, the Rab proteins.

The second mechanisms internalization (caveolar) has been clarified following the trafficking of some viruses that use caveolae to gain entry into the cells. Caveolae are small, flask-shaped invaginations of the plasma membrane with a size of about 50–60 nm that are rich in cholesterol and sphingolipids. Viruses initially associate with the cell membrane and then become trapped in relatively stationary caveolae. The subsequent intracellular uptake of caveolae leads to intracellular organelles that are distinct from classic endosomes; the presence of the protein caveolin in these organelles gave rise to the name caveosomes. In contrast to the dynamic nature of endosomes, caveolae are highly stable and are only slowly internalized. Another major difference is that the caveolar uptake does not lead to a decrease of pH and to a degradative pathway of their cargo, as in endosomal/lysosomal pathway.

It is clear that the different pathways of NPs internalization affect their delivery to different intracellular compartments and may affect their final destination. To obtain insight into the properties of drug delivery vehicles that direct their intracellular processing in brain endothelial cells, the intracellular traffic of fixed-size nanoparticles in an *in vitro* BBB model as a function of distinct nanoparticle surface modifications has been investigated. Previous observations [70] that latex particles with a diameter ≥500 nm are internalized by nonphagocytic B16 cells through caveolae, whereas particles up to 200 nm in diameter are efficiently taken up via clathrin-mediated endocytosis, were successively confirmed with Silica-Hydroxyl NPs on immortalized human brain capillary endothelial cell cultures [71]. In the investigation of Georgieva et al. [71] 500 nm particles were used uncoated, surface functionalized with cationic polymer polyethyleneimine (PEI), or with prion protein. The results suggested that uncoated NPs are internalized through caveolae; nanoparticles carrying a net cationic charge, accomplished by PEI, followed an adsorptive endocytotic route; and particles surface-modified with prion protein followed receptor-mediated endocytotic route. Therefore, the authors concluded that the NPs intracellular pathway is dictated by the particle surface characteristic, for a given size. Most interestingly, they found that the transcytotic potential is higher for the receptor-mediated and caveolar pathways and lower for the adsorptive-mediated.

However, the enhancement of brain delivery obtained with drug-loaded NPs, exploiting receptor- or caveolar- mediated transcytosis, is currently quantitatively limited in comparison with the free drug. Consequently also its clinical relevance is still scarce. If we compare the hydrodynamic size of most physiological proteins with that of NPs, we are confronting objects in the order of 5–10 nm size with those that, in most cases, are in the order of tenths of nanometers and commonly reach 100 nm, and more. Therefore, it is conceivable that the transcytotic traffic may become a bottleneck when transporting NPs. It is also predictable that smaller size NPs, closer to the size of physiological molecules, would be facilitated to enter the same intracellular transport pathways. Following the strategy of receptor-mediated transport, NPs made of gold, PLGA, chitosan, PAA, dendrimers, and liposomes have been functionalized, improving the delivery of drugs such as caspase inhibitors, endomorphin, tamoxifen, and tramadol and throwing the basis for treatment of neurological diseases [72–79].

Exploring and targeting new receptors that could be utilized for receptor-dependent endocytosis are likely to provide more efficient systems of brain drug delivery, mainly because these uptake mechanisms are relatively unaffected by lysosomal degradation.

#### 5.3.1. Lipoprotein Receptors

Apolipoprotein E (apoE) is a 34-kDa protein constituent of both very-low-density lipoprotein (VLDL) and high-density lipoprotein (HDL), which transports cholesterol and other lipids in the plasma and in the CNS [80, 81]. The lipoproteins complexes can be taken up in the brain through the recognition of apoE by specific receptors at the BBB, which include the low-density lipoprotein receptor (LDLR) and the LDLR-related protein (LRP).

Taking into consideration that LRP has been reported to be highly expressed on endothelial brain microvessels [82], receptor-mediated transcytosis using NPs functionalized to bind this target has been exploited in several ways.

First, by exploiting the preferential absorption of ApoE on some types of NPs when in serum. This feature has been described for PBCA NPs coated with polysorbate 80, indeed displaying the ability to cross the BBB *in vivo* in animal models [83–85] and for PEGylated PHDCA NPs, able to penetrate into brain endothelial cells *in vitro* [86].

Taking advantage of such opportunity, the formation of a stable ApoE decoration of NPs surface by covalent binding has been utilized to functionalize albumin-NPs or liposomes that showed the capacity of enhancing BBB passage *in vivo* in rodent models [87–89].

It should be questioned whether other apolipoproteins may be employed for NPs functionalization to confer on them the ability to enhance brain drug delivery. In order to answer this question, in an investigation of Kreuter et al. [90], PBCA NPs loaded with dalargin or loperamide were coated with different apo-lipoproteins, AII, B, CII, E, or J, coated or not with polysorbate 80, and then injected in mice and the drug effect on CNS was evaluated. The results showed that only NPs coated with apolipoprotein B or E were able to achieve an antinociceptive effect. This effect was significantly higher after polysorbate-precoating and apolipoprotein B or E-overcoating [90].

The region of apoE that is critical for interaction with the LDL receptor resides between amino acid residues 140 and 160. Several studies with synthetic peptides have investigated the structural features of the LDLR-binding sequence of ApoE [91, 92].

Laskowitz et al. [81] derived an apoE-mimetic peptide from amino acids 133–149, named COG133 (LRVRLASHLRKLRKRLL), which retains its biological activity *in vitro* and *in vivo*. This capacity is displayed also by a fragment of 26 a.a. from ApoE4 [93].

It has also been reported that the tandem dimer (141–155)_2_ is recognized by the LDLR, in contrast to the monomeric peptide (141–155) [94]. Also a shorter sequence of this peptide, the tandem dimer (141–150)_2_, retains this ability [95]. Probably as a consequence of these observations, sterically stabilized liposomes were functionalized only with ApoE tandem dimer peptide (141–150)_2_, but not with the monomer, and were shown to be efficiently taken up by rat brain capillary endothelial cells [96]. Interestingly, in spite of the theoretical premises based on receptor-mediated endocytosis, liposomes tagged with (141–150)_2_ were nonselectively internalized into cultured BBB cells, and clathrin- or caveolin-dependent endocytosis was not demonstrable [97].

However, in a successive investigation the ApoE-derived peptide monomer 141–150 was utilized by Re et al. [98] in comparison with the dimer, for the functionalization of liposomes entrapping a radioactive derivative of curcumin. The investigation showed a higher uptake of the drug by human endothelial cells and an enhancement of permeability of the drug across an *in vitro* model of the BBB made with the same cells, when using monomer-functionalized liposomes.

Apart from the amino acid sequence, other factors may affect the cellular uptake of these peptides [98]. These factors include the type of peptide, its cationic nature, its mode of exposure to the cell surface, the nature of the cargo, and the chemical linkage between the peptide and the cargo, other than the peptide surface density on the cellular uptake of nanoparticles [98, 99].

Finally, also Angiopep-2, a ligand of LRP, possesses a high brain penetration capability in both *in vitro* model of the BBB and *in situ* brain perfusion in mice [100]. PEG-PAMAM dendrimers functionalized using this peptide resulted in a high accumulation of a delivered gene into brain after intravenous administration into mice [101]; moreover, also functionalized polymeric NPs and carbon nanotubes (CNT) have been produced, showing the ability to reach glioma tumors in mice [102, 103].

#### 5.3.2. Transferrin Receptor (TfR)

The transferrin receptor (TfR) is the most widely studied receptor for BBB targeting. TfR is a transmembrane glycoprotein, consisting of two linked 90-kDa subunits, each one binding a transferrin molecule. The receptor is highly expressed on immature erythroid cells, placental tissue, and rapidly dividing cells, both normal and malignant [104]. Furthermore, it is expressed on hepatocytes and endothelial cells of the BBB. The role of the receptor is the regulation of cellular uptake of iron via transferrin, a plasma protein which transports iron in the circulation. Cellular uptake starts with the binding of transferrin to the transferrin receptor followed by endocytosis [105].

Iron-bound transferrin has a high affinity for the TfR; therefore, it has been used with success as a ligand for functionalization and targeting of liposomes to cultured brain endothelial cells [106, 107]. However, it is likely that that NPs decorated with transferrin would not be performant *in vivo*, since transferrin receptors are almost saturated in physiological conditions, because of the high circulating levels of endogenous protein [108]. Nevertheless, successful brain targeting using transferrin as a targeting ligand has been accomplished *in vivo* [109, 110]. Concerning the specificity of transferrin, a comparative study has been carried out between transferrin or lactoferrin, a multifunctional protein of the transferrin family widely represented in various secretory fluids, such as milk [111]. Lalani et al. [112] compared *in vivo* distribution in mice of PLGA NPs surface modified with the two proteins and showed a better performance to reach the brain in the case of lactoferrin.

In order to avoid the competition with endogenous transferrin circulating in blood, the use of monoclonal antibodies (mAbs) directed against transferrin receptors on the BBB has been suggested because they recognize different epitopes on the receptor [113]. For instance, OX26, 8D3, and R17217 mAb bind to TfR and have also been shown to undergo receptor-mediated transcytosis.

Among these, it is still debated which mAb is the best performing to be used for the decoration of NPs surface. The most well known is OX26, an antibody directed against the rat TfR, that has been successfully used in many brain targeting studies *in vivo* [114] and also *in vitro* using BBB cellular models [115]. However, Lee et al. [116] have shown that the OX26 monoclonal antibody is not an effective brain targeting molecule in mice. Moreover, they demonstrated that other two monoclonal antibodies, 8D3 and RI7217, directed against the mouse TfR, had a higher permeability of the mouse BBB *in vivo*, with respect to the brain uptake of the OX26 antibody, which was negligible. Additionally, they showed that RI7217 was more selective for the brain than 8D3, because this antibody was less taken up by the liver and kidney. It has been shown that RI7217 covalently coupled to human serum albumin is able to transport loperamide across the BBB [110]. More recently, it has been shown that also the chemical linkage of anti-TfR on the NPs surface could affect the crossing of BBB *in vitro* [117]. The authors compared liposomes covalently coupled with mAbs (obtained by reacting thiolated mAbs to phosphatidyl-ethanolamine maleimide inserted in the liposome bilayer) with liposomes decorated with biotinylated mAbs via an avidin bridge. The Authors found an higher cellular uptake and permeability across an *in vitro* BBB model made of immortalized human brain capillary endothelial cells of the liposomes covalently coupled with mAbs, suggesting that the covalent ligation could be preferentially chosen for NPs decoration.

### 5.4. Retrograde Transport

Transsynaptic retrograde transport could enable some types of nanocarriers to travel from peripheral nerve terminals to neuronal cell bodies in the CNS [118]. Studies in this regard have shown that NPs modified with PEI and other polyplexes display active retrograde transport along neurites but are unable to mediate efficient biological actions upon reaching the neuronal body [119].

### 5.5. BBB Breakdown

BBB breakdown occurs in neuroinflammatory diseases [120]. NPs can transiently and reversibly open the tight junctions located at the BBB and other sites, thus, increasing their paracellular permeability [121, 122]. In particular, blood-brain barrier disruption therapy is an intensive, effective way of sending medication to brain tumors [123].

Nevertheless, it is known that tight junctions can be opened only to a limited extent [124]; thus, only NPs smaller than about 20 nm can use this pathway to penetrate into the brain through the BBB.

### 5.6. Exploiting Monocyte/Macrophage Infiltration in the CNS

Monocyte/macrophage infiltration in the CNS plays a key role in neuroinflammation, as well as in lesion development and brain injury in neurological diseases such as multiple sclerosis (MS) and stroke [124–126]. Knowledge of active phases of cell infiltration during CNS disorders is important because anti-inflammatory treatments can target cell adhesion molecules and chemokines guiding cellular trafficking [127]. The monocyte infiltration through the BBB is also widely believed to play an important role in HIV infection of the CNS. The blood-brain barrier appears unaltered until the late stage of HIV encephalitis. HIV flux that moves toward the brain, thus, relies on hijacking with immune-activated leukocytes, mainly monocytes/macrophages from the periphery [128].

In this paper, BBB crossing by immune-activated macrophages appears to suggest possible strategies for future therapeutic developments employing NPs. This strategy could be realized at least in two ways: (1) by embedding NPs into activated monocytes used as Trojan horses to reach the brain (2) by designing NPs mimicking activated monocytes.

#### 5.6.1. Trojan Monocytes for NPs Delivery to the Brain

Nanoparticulate drug delivery systems have been used to avoid the RES clearance and achieve longer circulation time for enhanced tissue uptake. However, the enhancing of NPs phagocytosis by monocytes can be considered as an unconventional approach to deliver NPs-loaded drugs to the brain. After phagocytosis of NPs, monocytes, just like Trojan horses, may transport their cargo into the brain. Afergan et al. [129], taking this approach, embedded serotonin, a BBB impermeable neurological drug, into negatively charged liposomes and analyzed brain uptake in rats and rabbits. The performance of liposomal serotonin was significantly better, leading to two-fold higher drug concentration in brain than the free drug. Since treatment of animals by alendronate resulted with inhibition of monocytes but not of neutrophils, and with no brain delivery, the authors suggested that monocytes are the main transporters of liposomes to the brain. In spite of the fact that the clinical relevance of this investigation is limited, the Trojan monocyte approach provides a new possibility of more effective treatment of brain-associated inflammatory disorders, including multiple sclerosis and Alzheimer's disease, which are characterized with increased passage of immune cells across the BBB [130].

In other examples, in order to improve the delivery of contrast agents to the brain, iron oxide NPs administered intravenously *in vivo* have been shown to detect activated macrophage infiltration in multiple sclerosis either in the CNS of humans [131, 132] or rodent model [133–137], in stroke either in human [138, 139] or animal brain ischemia [140–143], in human or murine intracranial tumors [144, 145], in EAE [146–148], or in an ischemia/reperfusion long-lived rat model [149].

#### 5.6.2. NPs Mimicking Activated Monocytes

The therapeutic efficacy of drug-loaded NPs systemically administered depends on their ability to evade the immune system, to cross the biological barriers of the body, and to localize at target tissues. Parodi et al. [150] show that nanoporous silicon particles can successfully perform all these actions when they are coated with cellular membranes purified from leukocytes, avoiding being cleared by the immune system. Furthermore, they can communicate with endothelial cells through receptor-ligand interactions and transport and release a doxorubicin payload across an inflamed endothelial barrier *in vitro*. Also the accumulation of coated NPs in mice inoculated with murine B16 melanoma was enhanced compared with that of non coated NPs. Particle coating using leukocyte membranes led to an approximately twofold increase in particle density in the tumor.

Since several studies demonstrate increased passage of monocytes across the BBB in various pathological conditions, it is predictable that the research of NPs mimicking immune cells might be effective in brain-associated disorders and will receive a stimulus in this direction in the next years.

## 6. Factors Affecting NPs Brain Drug Delivery

### 6.1. NPs Diffusion inside the Brain Parenchyma

While this factor is likely not particularly relevant when brain drug delivery is sought after, it could be very important when the passage of the BBB by NPs is looked for, since the extracellular space (ECS) of the brain parenchyma could limit their diffusion or even preclude their entrance into the brain. The ability to achieve brain penetration with larger NPs is expected to allow more uniform, longer lasting, and effective delivery of drugs within the brain and may in particular find practice in the treatment of brain tumors, stroke, neuroinflammation, and other brain diseases where the BBB is compromised or where local delivery strategies are feasible.

Today it is well established that the ECS occupies a volume fraction of between 15% and 30% in normal adult brain tissue with a typical value of 20%, and that this falls to 5% during global ischemia [151, 152].

It is less obvious what the true size of the spaces between cells is. Small molecules such as inulin and sucrose diffuse through the ECS with a decreased diffusion coefficient with respect to water, suggesting the existence of limitation to their movements [153].

Possible sources of the limitations to the diffusing molecules are (a) the presence of cellular obstructions; (b) trapping of molecules in dead-space microdomains; (c) a viscous drag imposed by the macromolecules that compose the extracellular matrix or drag arising from the walls of the channels when molecules are large, such as in the case of NPs; (d) transient binding to cell membranes or extracellular matrix (e) nonspecific interaction with negative charges on the extracellular matrix when the diffusing molecule has adequate charge density.

A different type of model was proposed by Thorne and Nicholson [154] to explain the higher tortuosity experimentally measured for larger substances such as dextran and especially large synthetic quantum dot nanocrystals in the *in vivo* rat cortex. It was assumed that the quantum dots with a hydrodynamic diameter of 35 nm were close to the average width of the ECS, and depending on whether a planar or tubular model was adopted, the estimated ECS width was 38–64 nm.

In counter tendency to Thorne and Nicholson [154], Nance et al. [155] report that NPs as large as 114 nm in diameter diffused within the human and rat brain, only if they were densely coated with PEG. Using these minimally adhesive PEG-coated NPs, they estimated that human brain tissue ECS has some pores larger than 200 nm and that more than one-quarter of all pores are ≥100 nm.

In addition to ECS in CNS, there is also much interest in the possibility that perivascular spaces, fluid-filled channels surrounding arteries, arterioles, veins, venules, and possibly even microvessels offer potential pathways for rapid flow into and out of brain parenchyma. Measurements of perivascular space widths in mammals suggest that their typical dimensions may be at least 2 orders of magnitude greater than the neocortical ECS width (e.g., arteriole perivascular spaces have been reported in the range of 5–10 *μ*m or larger (in rodents and humans)).

### 6.2. Effect of Protein Corona

Different physicochemical properties, not only size, may determine the ability of nanoparticles to reach the brain. The situation is further complicated by the formation of a so-called “corona” of biomolecules on the surfaces of nanoparticles, the composition of which may vary depending on the route of exposure, and on the physicochemical surface properties of NPs. The acquisition of a corona of biomolecules on the surface of NPs may also determine whether NPs are able to cross from one compartment to another and whether they are taken up effectively by cells or not [156].

In practical situations in which nanoparticles interact with living organisms, the nanoparticle surfaces are initially exposed to a biological fluid, such as blood, depending on the route of administration. For instance, NPs injected intravenously would be exposed to blood plasma, containing an excess of proteins and many other complex biomolecules, which bind competitively with the surface of the nanoparticles. 

The current idea is that “bare” nanoparticles do not exist *in vivo*, because they are immediately modified by the adsorption of blood proteins with higher affinities for the particle surface, forming a more or less tightly bound layer (the so-called hard corona) and a weakly associated mobile layer (the so-called soft corona). When this issue was investigated, it has been found that typical coronas contain a limited number and types of molecules gathered from the blood, in spite of the fact that biological fluids contain thousands of proteins [157]. Moreover, it should be noted that the corona may also play a role in other undesirable effects of NPs in living systems such as complement activation and blood clotting and may not necessarily play a role only in cellular uptake.

It should be pointed out that none of the *in vitro* experiments conducted to study the process of NPs translocation to the brain takes into account the surface modification of NPs inside the blood and how can this affect the crossing of the BBB. This issue needs much further investigation. Moreover, NPs once internalized by endothelial BBB cells, they may also exit towards the brain covered with different biomolecules depending on that they have undergone endocytosis/transcytosis/exocytosis, and, thus, exert additional effects (e.g., toxicity) on neuron. Also this issue has not yet been investigated.

### 6.3. BBB Alterations in Neurological Diseases

The peculiar physiological features of the BBB affect the extent of drug permeability and in most cases extremely restrict the amount of pharmaceuticals taken up by the brain. It should be pointed out that the anatomy and organization of the BBB are altered in a number of pathological conditions, and these changes could have consequences onto the crossing of the barrier by physiological circulating molecules, drugs, and NPs. It should be also pointed out that changes are not the same for all the diseases: the variability is high; thus, it is hard to predict the effects on any particular drug. In general, the BBB alterations could cause shifts in the dosage, efficacy, and side effect of commonly used drugs.

For instance, the effect of hypoxia-ischemia on the barrier has been extensively investigated. Hypoxia-ischemia starts a series of events, which lead to increased BBB permeability, possibly due to disruption of tight junctions and mediated by signaling molecules such as cytokines and nitric oxide [158]. It is expected that the passage of macromolecules across the BBB is increased under this condition. It is therefore significant that nanostructured erythropoietin may exert a neuroprotective action against hypoxia-ischemia in animal models [159].

A variety of evidence demonstrates that the BBB is also compromised in septic encephalopathy, where albumin [160] has been shown to enter brain parenchyma from the circulation in rodents.

Also a series of neurologic inflammatory diseases, including HIV-associated dementia and multiple sclerosis, strongly alter the integrity of the BBB with consequent migration of leukocytes into the brain [161, 162]. The migration has been shown to trigger signals leading to loss of tight junctions molecules and to opening of the BBB [163].

Although there is no evidence in humans or animal models for a massive disruption of the BBB in Alzheimer disease (AD), there is evidence of a decreased glucose and oxygen use by the AD brain. If the decrease represents a defect in the flow across the barrier or in response to a decreased requirement by the CNS is unclear, however, it is consistent with the AD environment promoting barrier cell secretions that have detrimental effects on cognition [164]. What is clear is that BBB endothelial cells bind and internalize *β*-amyloid peptide (A*β*), where the peptide mostly remains adherent to or internalized by the cells. A*β* induces chemokine secretion, monocyte trafficking, decreased proliferation, altered permeability, and altered nitric oxide synthase activity. Micro-lesions representing limited protein leakage at capillaries have been demonstrated in some animal models of AD [165]. Interestingly, one of the few drugs available for the treatment of AD, the NMDA receptor antagonist memantine, protects against BBB disruption. 

### 6.4. Size

An underestimated issue is whether or not the size of NPs designed for reaching the brain drug delivery makes any difference. Different investigations have been carried in the attempt to clarify this issue. Sarin et al. [166] after having intravenously administered functionalized PAMAM dendrimers with size less than approximately 12 nm found that NPs were able to traverse pores of the blood-brain tumor barrier of RG-2 malignant gliomas, while larger ones could not.

Sonavane et al. [167] studied tissue distribution of colloidal gold nanoparticles of different sizes (15, 50, 100, and 200 nm) after intravenous administration in mice. The number of NPs which entered the brain after 24 h was inversely dependent on the size and for 15 nm gold NPs was 500-fold higher than for 100 nm NPs, while was very low for 200 nm NPs. However, the total amount of gold, which is proportional to total volume of NPs entered, thus, to the payload of drug in the case of drug-loaded NPs, was similar for 15 and 50 and only 30% lower for 100 nm. Oberdörster et al. [168] generated solid ultrafine particles of size around 36 nm made of graphite that were administered by inhalation by rodents. The authors concluded that the CNS can be targeted by airborne NPs of this size via the olfactory nerve from the olfactory mucosa.

Recent evidence suggests that the transit through the gastrointestinal tract may strongly influence the subsequent access of NPs into the brain. Hillyer and Albrecht [169] studied the gastrointestinal uptake and tissue/organ distribution of 4, 10, 28, and 58 nm diameter metallic colloidal gold NPs. The gold NPs were administered orally, and gold concentration in various tissues/organs, brain included, was determined after 12 h. The author found that the total amount of gold uptaken in brain was similar for 4 and 58 nm particle size. Also Schleh et al. [170] investigated the influence of size and surface charge of gold nanoparticles on the absorption across intestinal barriers and accumulation in brain after oral administration. The authors utilizing NPs from 1.4 to 200 nm found the highest accumulation in secondary organs for 1.4 nm particles, while the highest brain accumulation was recorded in the case of 18 nm NPs. These data suggest that the route of administration of NPs into the body is of pharmacological and clinical importance and that NPs may cross the BBB whichever the initial route of administration.

## 7. Neurotoxicity Issues

Even if the use of engineered NPs represents one of the main hopes for innovative pharmacological strategies in neurology [171], it is important to mention that the BBB represents a mechanism of defense of CNS against potentially neurotoxic molecules and structures, NPs included. *In vivo* and clinical data evaluating the toxic effects of NPs on neural cells are still scarce, and it is still difficult to extrapolate the results obtained on *in vitro* models to the actual situation *in vivo* [172], given that the application of NPs to the CNS is at a nascent stage.

It should be pointed out that different types of NPs, showing promising features for *in vivo* applications at the beginning, were successively discarded after having demonstrated their toxicity when utilized *in vivo*. This is the case, for instance, of quantum dots and carbon nanotubes that proved to be toxic *in vivo* [173], and for this reason, their prospective use in medicine will be likely limited to *in vitro* diagnostics. Other cases of NPs-related neurotoxicity have been reported, and neurological effects were described in mice exposed to SiO_2_ and MnO NPs [174]. *In vivo* experiments showed that other NPs also have some neurotoxicity by causing transient microglia activation and induction of TLR-2 promoter activity in transgenic mice [175].

For these reasons, the benefit-risk balance deriving from theuse of NPs intended for treatment of CNS diseases should be carefully evaluated for each type of new engineered NPs. When assessing the possible neurotoxicity of NPs, it should be pointed out that beyond of the core structure, also surface functionalization, often employed for targeted delivery, can significantly alter the biological response and induce neurotoxicity of otherwise safe particles [176]. Therefore, this aspect also needs to be taken into consideration. Moreover, it should also be stressed that toxicity could depend by the opening of tight junctions of the BBB endothelia, induced by NPs. For instance, an *in vitro* study by Olivier et al. [177] showed that PBCA NPs induced a permeabilization of a BBB model, which was presumably attributed to the toxicity of the carrier.

The most reliable data on the safety of NPs towards CNS have been reported for liposomes and iron oxide NPs. Liposomes are known from the 60s and are generally low toxic because they are composed of naturally occurring lipids [178]. Iron oxide-based NPs are also believed to be scarcely toxic to the CNS [179, 180].

Another source of neurotoxicity could arise from the functionalization of NPs with cationized proteins [181]. Toxic effects have been detected only when such proteins are administered to “heterologous” animals. In view of this, human proteins or recombinant humanized proteins should be used for cationization and subsequent applications in humans. Moreover, since the conjugation of proteins to polyethylene glycol has been shown to decrease their immunogenicity, PEGylation of cationized molecules may be an alternative to minimize the immunogenic potential of these molecules.

## 8. Future Directions

Given the importance of finding new drug delivery systems for treatment of CNS diseases it is conceivable that new strategies will be developed in the next future.

Since several studies demonstrate increased passage of monocytes across the BBB in various pathological conditions, the synthesis of NPs mimicking immune cells might be effective in brain-associated disorders, and it is therefore predictable that the research will receive a stimulus in this direction in the next years. It is also hypothesizable that NPs designed to mimic the molecular interactions occurring between inflamed leukocytes and endothelium should possess selectivity toward diverse host inflammatory responses, for instance, the incorporation of inflammation-sensitive sensors such as integrin lymphocyte function-associated antigen (LFA)-1 I domain, to mimic activated leukocytes for the targeting of inflamed tumor microenvironments, Or CCR2, since cerebral microglia can recruit an increased number of activated circulating monocytes into the brain in response to elevated cerebral monocyte chemoattractant protein (MCP)-1.

Other possibilities are to exploit the absence or at least the high permeability of some BBB regions. The BBB is present in all brain regions, with the exception area postrema, median eminence, neurohypophysis, pineal gland, subfornical organ, and lamina terminalis. The endothelial cells present in capillaries of these brain areas have fenestrations that allow diffusion of molecules. The most extended area where, presumably, a more extensive passage of NPs could be possible is occupied by the leptomeningeal space, which is the space occupied by Cerebrospinal fluid (CSF), including all spaces continuous with the subarachnoid space, such as perivascular spaces and ventricles [182]. The data available to date suggest that many macromolecules and nanoparticles can be delivered to CNS in biologically significant amounts.

Moreover, the perivascular spaces which are in continuity with leptomeningeal space penetrate into the parenchyma provide an unexplored avenue for drug transport deep into the brain via CSF. The flux of the interstitial fluid in the CNS parenchyma, as well as the macro flux of CSF in the leptomeningeal space, is believed to be generally opposite to the desirable direction of CNS-targeted drug delivery. The final outcome will depend on the NPs behavior, which has not been studied yet. It is therefore conceivable that efforts in this direction will be carried out in the next future, in order to exploit alternative administration routes.

## 9. Conclusions

The number of deaths due to neurological or neurodegenerative diseases is those of a world war, with connected huge socioeconomical problems and costs. The treatment of such diseases is hampered by the presence of BBB, insurmountable by most available and future potentially effective drugs. Therefore, the discovery and development of novel drug delivery systems for the treatment of such diseases is a major challenge for both the academic and pharmaceutical community. Nanotechnology represents an innovative and promising approach. Currently, several types of NPs are available for biomedical use with different features and applications facilitating the delivery of neuroactive molecules such as drugs, growth factors and genes, and cells to the brain. NPs offer clinical advantages for drug delivery such as decreased drug dose, reduced side effects, increased drug half-life, and the possibility to enhance drug crossing across the BBB. However, the enhancement of brain delivery obtained with drug-loaded NPs, although very promising, is still quantitatively limited in comparison with free drugs. Consequently, with very few exceptions, NPs are not yet a viable solution for pharmacology, requiring enhancements of one order of magnitude or more.

Further investigations are necessary for a better comprehension of the mechanisms which manage this different NPs-mediated transport of the drugs to the brain. However, the strong efforts to allow the translation from preclinical to concrete clinical applications are worth of the necessary economic investments.
